# Piezo-Resistive Flexible Pressure Sensor by Blade-Coating Graphene–Silver Nanosheet–Polymer Nanocomposite

**DOI:** 10.3390/nano13010004

**Published:** 2022-12-20

**Authors:** Zheng Kang, Xiangmeng Li, Xiaodong Zhao, Xiaoqiang Wang, Jian Shen, Huifen Wei, Xijing Zhu

**Affiliations:** 1 Shanxi Provincial Key Laboratory of Advanced Manufacturing Technology, North University of China, Taiyuan 030051, China; 2 State Key Laboratory for Manufacturing Systems Engineering, Xi’an Jiaotong University, Xi’an 710049, China

**Keywords:** piezo-resistive sensor, flexible sensor, imprinting, blade coating, graphene, silver nanosheet, polymeric nanocomposite

## Abstract

The demand for flexible pressure sensors in wearable devices is dramatically increasing. However, challenges still exist in making flexible pressure sensors, including complex or costly fabrication processes and difficulty in mass production. In this paper, a new method is proposed for preparing the flexible pressure sensors that combines an imprinting technique with blade-coating of a graphene–silver nanosheet–polymer nanocomposite. The piezo-resistive type flexible pressure sensor consists of interdigital electrodes and nanocomposite as a sensing layer, as well as a micropillar array structure. The morphology of the sensitive layer of the sensor is characterized by scanning electron microscopy (SEM). The response performance, sensitivity, and stability of the sensor are investigated. The test results show that the initial resistance of the pressure sensor is only 1.6 Ω, the sensitivity is 0.04 kPa^−1^, and the response time is about 286 ms. In addition, a highly hydrophobic wetting property can be observed on the functional structure surface of the sensor. The contact angle is 137.2 degrees, revealing the self-cleaning property of the sensor. Finally, the prepared sensor is demonstrated as a wearable device, indicating promising potential in practical applications.

## 1. Introduction

With rapid development of soft robots [1], human-computer interactions [2], smart homes [3,4], and medical health monitoring [5], flexible electronic products such as electronic skin [6], wearable devices [5], and on-site detection sensors [7,8] have become hot topics. These flexible electronic devices are highly desirable with accurate sensing abilities. However, traditional sensors based on metal thin-films or semiconductor materials are difficult to adapt to the irregular curved surfaces of human bodies or soft robotic manipulators due to their low deformation. Researchers have tried to improve the flexibility of these sensors by adopting some serpentine film-like microstructures [9]. Recently, new flexible or stretchable sensors are emerging, including pressure sensors [10], temperature sensors [11], humidity sensors [12], and so on. Among them, the flexible pressure sensor has been particularly attractive for a wide range of application prospects. For example, flexible pressure sensors can sensitively perceive the human pulse, heartbeat, and motion state for medical health monitoring [13] and tactile perception [14]. Additionally, the flexible sensors can be also applied to soft robots [15]. However, it is a great challenge to achieve low-cost, large-scale, and high-performance flexible pressure sensors. Therefore, it is desirable to develop new sensor fabrication approaches.

Numerous nanomaterials have been widely used in flexible pressure sensors because of their excellent electrical properties, such as graphene [16], carbon nanotubes [17,18], and other carbon-based nanomaterials, metal-based nanowires, nano-sheets, and two-dimensional carbides and nitrides (MXenes) [19]. Researchers have tried many different materials as flexible substrate materials, including paper, silk [20], poly(ethylene terephthalate) (PET), polyimide (PI), polydimethylsiloxane (PDMS), and so on [21]. The detection of pressure can be achieved based on the response of resistance [22], capacitance, piezoelectricity, and triboelectric effect to the pressure [23]. Furthermore, to enhance the sensitivity and response performance of the flexible pressure sensors, researchers have proposed many kinds of sensitive materials and structures. For example, helical and serpentine structures were used to improve the ductility of metal film electrodes or sensitive materials for flexible sensors [24,25]. Meanwhile, regular array micropillar structures of cylinders, cones, and replica structures from irregular microstructures such as sandpaper or plant leaves were employed to improve the sensitivity of flexible sensors [26]. Importantly, researchers have also developed a great many approaches to achieve these structures, including lithography, etching, screen printing, electrospinning, inkjet printing, and so forth. Although great progress has been made in the research on materials, structures, and fabrication, great challenges remain in sensor fabrication due to its complex processes.

In recent years, flexible sensors based on polymeric composite materials have become highly promising in the application of wearable electronics. For instance, highly stretchable hydrogels have similar mechanical properties as human skin, and in particular, ionic hydrogels endow flexible sensors with excellent response performance [27]. Therefore, such hydrogels have attracted wide attention from researchers. Three-dimensional (3D) printing techniques have been proposed to achieve complex patterns of hydrogels. However, there exist some disadvantages of hydrogel-based sensors; e.g., the resulting hydrogel-based materials are of poor conductivity and greatly rely on there being a humid environment.

Herein, we propose a novel type of polymeric nanocomposite based flexible sensor that demonstrates good conductivity and response performance. Meanwhile, by adopting relatively simple and cost-effective processes, such flexible sensors can be achieved and can reveal high hydrophobicity. Templates of silicone rubber ESSIL 296 are fabricated by imprinting to prepare a micro-hole array structure and a micro-channel for interdigital electrodes. Then, interdigital electrodes and a pressure-sensitive functional layer of the flexible pressure sensor can be achieved by blade-coating. The sensitive functional layer is made of reduced oxide graphene (RGO), silver nanosheet (AgNS), and PDMS. The sensing performance and surface wetting properties are investigated. Finally, the flexible pressure sensor as prepared is demonstrated as a wearable device.

## 2. Materials and Methods

### 2.1. Materials

During the fabrication of the flexible pressure sensor, ESSIL 296 (a mixture of polymethylsiloxane and silicon dioxide) was used as the sensor substrate, PDMS (Sylgard184, Dow Corning, Midland, MI, USA) was used as the functional layer polymer nanocomposite material, silver nanosheet (AgNS) with a sheet diameter of about 5 μm (XF Nano Co., Ltd., Nanjing, China), and an aqueous dispersion of high-concentration reduced graphene oxide (RGO) (XF Nano Co., Ltd., Nanjing, China) were used as purchased.

### 2.2. Preparation of Sensitive Layer Material

To fabricate the sensitive layer of the flexible sensor, PDMS, AgNS, and RGO dispersion were mixed uniformly in a mass fraction of 10:10:1. First, high concentration graphene dispersion was treated in an ultra-sonication bath for 15 min to ensure uniform dispersion. Then, the RGO dispersion and the AgNS were weighed and mixed thoroughly with ultra-sonication treatment for 10 min. Next, the PDMS precursor was added to the mixture, and ultrasonic treatment was continued for 15 min. When the ultrasonic treatment was finished, the PDMS curing agent was added to the mixture under rigorous stirring for about 10 min to produce a viscous state. In this manner, a uniform mixture of slurry of polymeric nanocomposite could be obtained as the sensitive material.

### 2.3. Fabrication Process of Flexible Pressure Sensor

**Figure 1:**
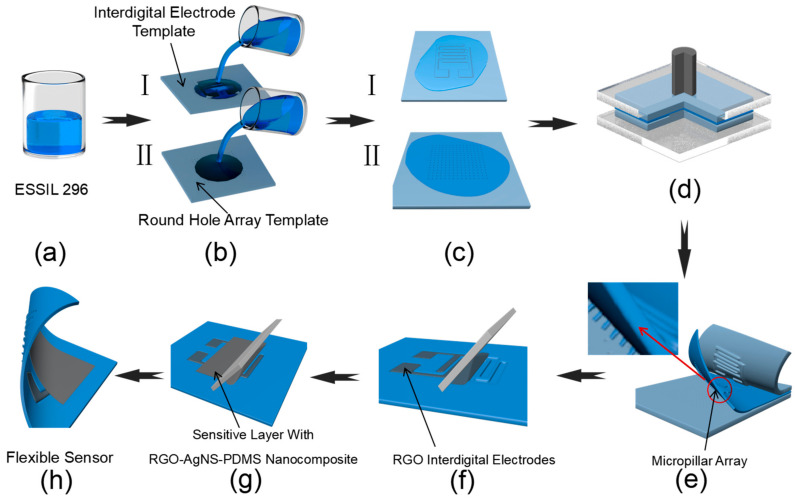
Schematic illustration of sensor fabrication procedure. (**a**) Stirring of ESSIL 296 with curing agent with a mass fraction of 10:1; (**b**) Spin coating of ESSIL 296 mixture on interdigital electrode template (**b-I**) and the round-shape hole array microstructured template (**b-II**); (**c**) Placing together of the interdigital electrode template and the micropillar array template in a vacuum chamber for 8 min to further degas; (**d**) Alignment of (**c-I**) and (**c-II**) flat surfaces with ESSIL 296, and clamping both templates together with two pieces of glass slides under a certain pressure, prior to curing in an oven; (**e**) Peeling the cured ESSIL 296 from the interdigital electrode template and the round-shape hole array template; (**f**) Blade-coating RGO ink into the interdigital electrode channel; (**g**) Blade-coating of RGO–AgNS–PDMS nanocomposite to prepare for a sensitive layer on the surface of the interdigital electrodes. (**h**) Illustration of the flexible sensor.

**Figure A1:**
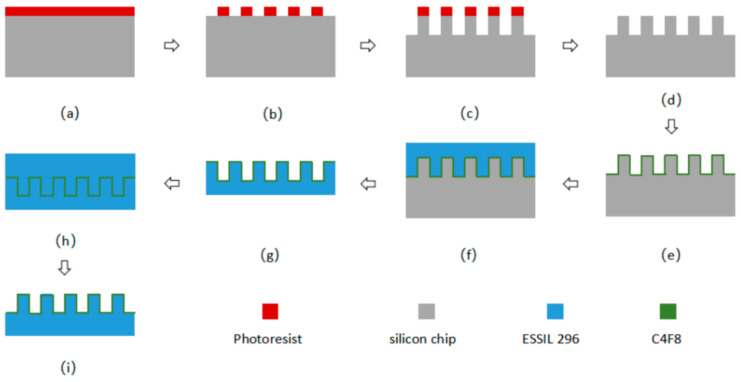
Schematic illustration of fabricating the ESSIL296 microhole array template. (**a**) Spin-coating of photoresist AZ4620 on cleaned silicon wafer; (**b**) Photoresist patterning by UV exposure and development; (**c**) ICP/RIE etching of microhole array structures on silicon wafer; (**d**) Removal of photoresist on silicon structures; (**e**) Hydrophobic treatment with C_4_F_8_; (**f**) Spin-coating of ESSIL 296 on microhole array structure of silicon wafer, template imprinting and curing; (**g**) Peeling of cured micropillar array structures of ESSIL 296, as well as hydrophobic treatment with C_4_F_8_; (**h**) Spin-coating of ESSIL 296 on the hydrophobic-treated ESSIL 296 template and curing; (**i**) Peeling of ESSIL 296 microhole array structures and another hydrophobic treatment.

Figure 1 shows the fabrication procedure of the flexible pressure sensor proposed. First, 30 g of ESSIL 296 with a 10:1 ratio of basic components and curing agent was mixed together by rigorously stirring prior to being degassed in a vacuum chamber (Figure 1a). Then, the ESSIL 296 mixture was spin-coated onto the interdigital electrode template and the round-shape hole array template (Figure 1b), respectively. The template was fabricated according to the scheme of procedure as shown in Figure A1 (Appendix A). Then, the two resulting templates of ESSIL 296 were placed together face-to-face in a vacuum for further degassing (Figure 1c). After that, the flat surfaces coated with ESSIL 296 on the two templates were closely adhered to each other, and a certain pressing force was applied from both sides to promote bonding of the two templates (Figure 1d). Finally, the bonded sample was placed in an 85 °C oven for 30 min; thus, a film sample could be obtained with microstructures on both sides.

The film sample with two sides of microstructures (one side with a micro-channel of interdigital electrodes and the other side with a micropillar array structure) was peeled off the templates (Figure 1e). Then, about 1 mL of high-concentration RGO ink was applied to the surface by blade-coating with interdigital electrodes. Upon repeating blade-coating and drying on a hotplate, interdigital electrodes with an RGO structure were obtained (Figure 1f). After that, the residual RGO ink on the surface was cleaned with a dust-free cloth soaked in ethanol to avoid short circuit of the electrode. Then, the RGO–AgNS–PDMS nanomaterial composite prepared in Section 2.2 in the amount of 1 mL was used to fabricate the sensation layer. Briefly, the slurry nanocomposite could be deposited into the template structures by blade-coating over the desired area and cured in an oven at 90 °C for 2 h (Figure 1g). Thus, a conductive and pressure-sensitive layer about 100 μm in thickness was prepared on the surface of the above prepared interdigital electrodes. The mixing ratio of the composites could be adjusted to achieve an optimized sensing performance. Finally, a layer of PDMS was spin-coated on the surface of the sensor device for packaging (Figure 1h).

### 2.4. Morphology Characterization and Surface Wettability Test

The structural morphology of RGO, AgNS, and PDMS composites was observed by field emission scanning electron microscope FE-SEM (SUPRA-55, ZEISS, Oberkochen, Germany). The microstructures of the sensor and interdigital electrodes were characterized using a confocal microscope (OLS5000-SFA, Olympus, Tokyo, Japan). Then, the static and dynamic surface wettability of ESSIL 296 samples were characterized for surfaces with or without micropillar array structures. At the same time, a high-speed camera (FASTCAM NOVA S9, PHOTRON Ltd., Tokyo, Japan) was employed to record the sliding and rolling process of a 10-μL water droplet on the 45° tilting surface with micropillar array structure. The parameters of the high-speed camera included a shutter speed of 1/frame sec, and a shooting speed of 1000 fps (frames per second).

### 2.5. Performance Test of the Flexible Sensor

**Figure A2:**
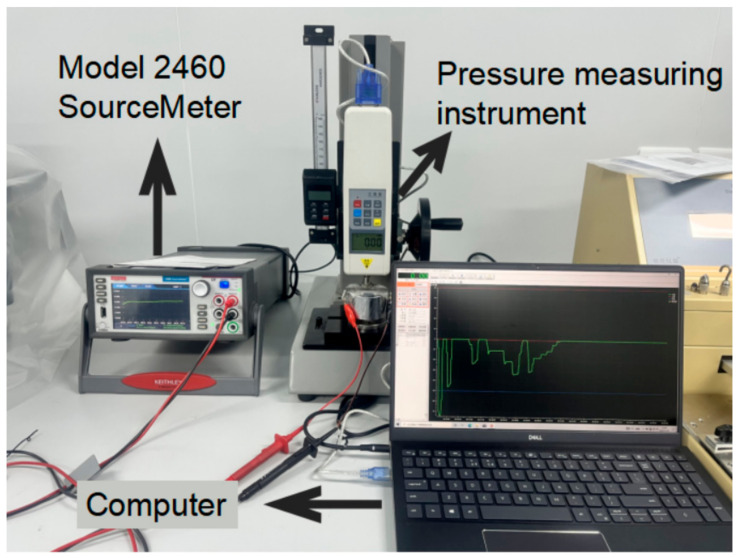
Photos of experimental set-up for testing the flexible pressure sensor.

To investigate the performance of the prepared sensor, a High Current Interactive Source Meter Model 2460 (Keithley Instruments, Solon, OH, USA) was used with data acquisition frequency of 12 points per second. A digital display push–pull meter (HP-50, Edberg Instruments Co., Ltd., Yueqing, China) was employed to apply load to the sensor, and the data were obtained with a sample frequency of 12 points per second. The experimental set-up for testing the sensor performance can be seen in Figure A2 (Appendix A). To investigate the response, stability, and sensitivity of the flexible pressure sensor, we applied loads to the flexible pressure sensor from 0 to 15 kPa with different loading rates with data recorded. In total, four groups of data were analyzed, and the response time was obtained for different loading rates.

Furthermore, three modes of pressure loading approaches were implemented to figure out the comparable response of the sensors. In the first approach, pressure loading was applied continuously from 0 to 40 kPa until a stable pressure was reached for each single 5 kPa step; this experiment was repeated 5 times. For the second approach, pressure loading was applied from 0 to 5 kPa and unloaded to 0, then the pressure was increased again until it reached 40 kPa, and this process was also repeated 5 times. Then, for the third approach, pressure was applied from 0 to 40 kPa and started to unload after stabilization, down to 35 kPa and to 5 kPa successively. This process was also repeated five times. The aim of the different loading modes was to test whether the resistance changed consistently with the pressure loading under different modes. Finally, the sensitivity and stability characteristics were analyzed by recording data.

## 3. Results

### 3.1. Working Mechanism of the Sensor

**Figure A3:**
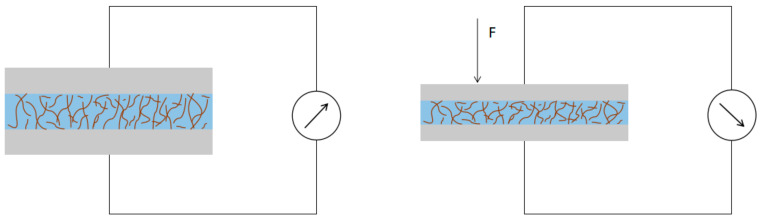
Schematic illustration of the piezo-resistive flexible sensor before and after loading pressure. By applying pressure loading *F* on the conductive polymer, more conductive paths can be generated, thus leading to a decrease in resistance.

The working principle of the prepared sensor is based on the permeation theory and the tunneling current effect [28,29], as the conductive nanocomposite material was prepared by adding conductive nanomaterials into the silicone rubber, where a conductive network path would be generated in the nanocomposite. The generation of a conductive network path is sensitive to application of pressure and has a low resistance. By increasing the loading pressure, the internal conductive path will become shorter and thus lead to a decrease in resistance. The deformation and corresponding output of the sensor before and after pressure are shown in Figure A3 (Appendix A). The corresponding mechanism can be described as in the following equations [28]:

(1) $$\begin{array}{c} J=J_{0}\left\{\bar{\varphi }e^{-A\sqrt{\bar{\varphi }}}-\left(\bar{\varphi }+eV_{e}\right)e^{-A\sqrt{\bar{\varphi }}+eV_{e}}\right\}\\ J_{0}=\frac{e}{2\pi h{\left(\Delta s\right)}^{2}}\\ A=\frac{4\pi \Delta s}{h}\sqrt{2m_{e}} \end{array}$$

where *J* represents the tunnel current density, *e* and *m_e_* represent the charge and number of electrons, *h* is Planck’s constant, *V_e_* is the applied voltage across the thin polymer layer, and $\bar{\varphi }$ is the average energy gap, which has two roots at two positions of *s*_1_ and *s*_2_ with a distance of Δ*s* = *s*_2_ − *s*_1_.

### 3.2. Dispersion of the Functional Layer

To obtain an optimized sensitive layer, six groups of nanomaterial application were explored, as listed in Table 1. The results show that PDMS has good flowability in the liquid state and that the PDMS mixture with precursor and curing agent will not cure at room temperature. However, the toughness of PDMS after curing was worse than ESSIL 296, so it was easy to tear. On the contrary, ESSIL 296 has poor flowability and has higher viscosity in the liquid state. Being mixed with the curing agent, ESSIL 296 mixture would be solidified at room temperature after a relatively long time, but it retained good toughness and was not easy to tear.

Since ESSIL 296 was selected as the substrate material, ESSIL 296 was thought to be applicable as a matrix polymer for the sensitive nanocomposite layer. On the one hand, it was found that the nanocomposites were not conductive when a small amount of RGO dispersion was added to PDMS or ESSIL 296. On the other hand, if the amount of RGO was increased, then the RGO and PDMS or ESSIL 296 could not be effectively bonded together. In that case, it was not suitable to use the RGO and ESSIL nanocomposite as the sensation material. It is notable that RGO is a material of low density, and therefore its mass is far less than that of silicone rubber, leading to a large volume. Alternatively, AgNS was applied with both PDMS and ESSIL 296. As a result, RGO could be well dispersed with PDMS or ESSIL 296, but the electrical conductivity was poor and unstable, and AgNS was not uniformly dispersed in silicone rubber, especially for ESSIL 296.

**Figure 2:**
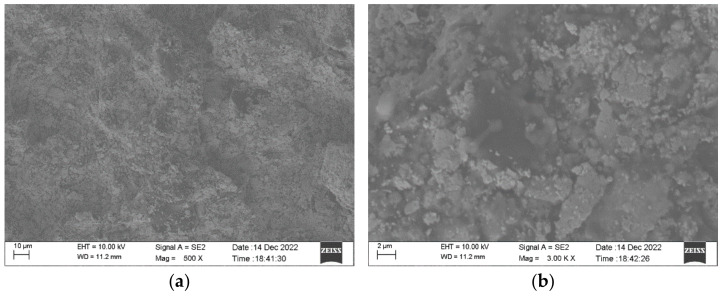
Morphology of RGO–AgNS–PDMS nanocomposites. (**a**) SEM images of RGO–AgNS–PDMS nanocomposites at a magnification of 500×; (**b**) SEM images of RGO–AgNS–PDMS composites at a magnification of 3000×.

Therefore, by combining the advantages of different matrix polymers and nano-fillers, RGO, AgNS, and PDMS were eventually chosen for preparing the nanocomposite. By adjusting the ratio of these components, polymeric nanocomposites with low resistance, good viscosity, and stable performance could be obtained. Figure 2 shows the morphology of a sensitive layer of the RGO, AgNS, and PDMS nanocomposite observed at different levels of magnification. As can be seen, RGO and AgNS are well dispersed without obvious aggregates. In addition, AgNS is wrapped by PDMS, indicating that AgNS would be more robust and embedded in the polymer matrix. Therefore, we can also verify that the nanocomposite in PDMS could have good stability. By adjusting the proportion of nanofillers in PDMS, the resistance of the nanocomposite could vary from several tens of ohms to hundreds of ohms. Such resistance is smaller than those polymers with resistance of thousands or even tens of thousands of ohms [30,31,32].

### 3.3. Morphology of the Sensor Substrate

**Figure 3:**
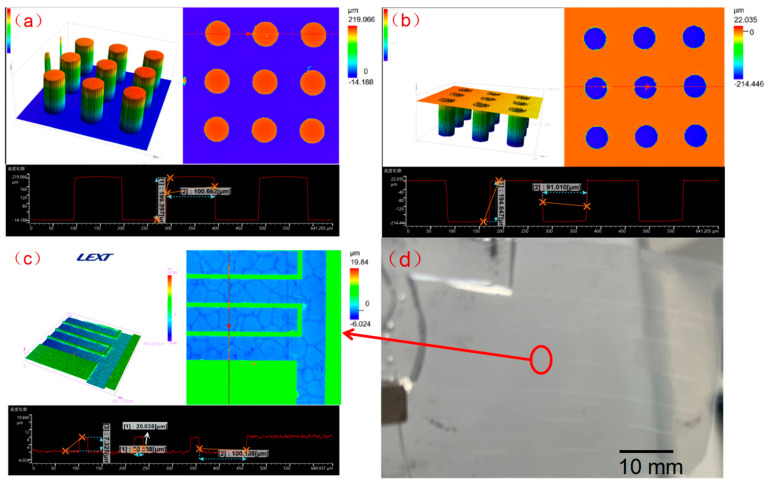
Confocal microscope images of surface topography of the sensor. (**a**) Microscope image of micropillar array structure; (**b**) Microscope image of micro-hole array structure as a template for replicating the micropillar array structure in (**a**); (**c**) Microscope image showing the channel structures for template fabrication of the RGO interdigital electrodes; (**d**) Photographs of ESSIL 296 thin films with channels for templating fabrication of interdigital electrodes, with the circle indicating the channel structure, and micropillar array structures on other surface of the film.

Figure 3 shows the morphology of the sensor observed by confocal microscope. Figure 3a shows the surface morphology of the micropillar array structure. It can be seen that the molded structure of the micropillars is complete, with a height of 200 μm and a diameter of 100 μm. These micropillar array structures resulted from replicating the round-hole microstructure as shown in Figure 3b. Figure 3c shows the channel morphology of the interdigital electrodes resulting from imprinting. The width of the interdigital electrode was about 100 μm, while the gap was 20 μm, and the channel depth was about 8 μm. As can be seen in Figure 3, the surface microstructure could be successfully replicated by imprinting technology in a high throughput manner.

### 3.4. Response Behavior of the Flexible Sensor

**Figure 4:**
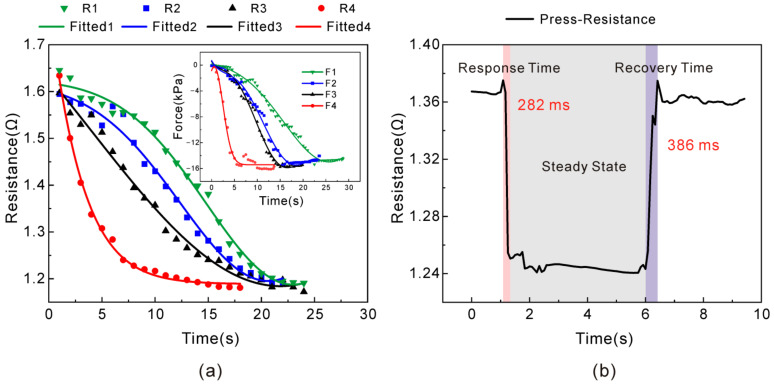
Correlation of resistance response with time of sensor during pressure loading. (**a**) Pressure loading application to 15 kPa by four loading modes, indicating the variation of resistance with increasing the pressure; (**b**) Curves show the response time and the recovery time of sensor during loading and unloading processes, respectively.

Figure 4a depicts four pressure loading modes for multiple time points; they finally reached a stabilized response at 15 kPa, and four resistance curves of R1–R4 were correspondingly obtained. It can be seen that as the load increases at different speeds, it gradually stabilized at 15 kPa, and the resistance gradually decreased to a similar value of about 1.2 Ω, indicating the stability of the sensor with the pressure change. Meanwhile, the resistance change decreased as the pressure was increasing, which was not affected by the loading speed. Notably, the sensor was tested repeatedly more than 60 times, and the final output values of resistive response almost approached the same, revealing good repetition characteristics. Figure 4b shows that when a certain pressure was applied to the sensor and unloaded within a very short duration, the response time of the sensor was 282 ms and the recovery time was found to be 386 ms, indicating that the sensor has a fast response. However, the recovery time was slightly longer than the response time, because the RGO–AgNS–PDMS polymer nanocomposite was a kind of elastic material with mechanical hysteresis. Therefore, it would take some time for the internal conductive path to relax.

### 3.5. Sensitivity and Stability Test of the Sensor

The sensitivity of the prepared piezo-resistive pressure sensor can be described as

(2) $$S=\frac{R_{1}-R_{0}}{R_{0}\Delta p}=\frac{\Delta R}{R_{0}\Delta p}$$

where *S* is sensitivity (kPa^−1^); *R*_0_ the initial resistance of the sensor (Ω); *R*_1_ the resistance value in applying the pressure (Ω); ∆*p* the applied load (Pa).

**Figure 5:**
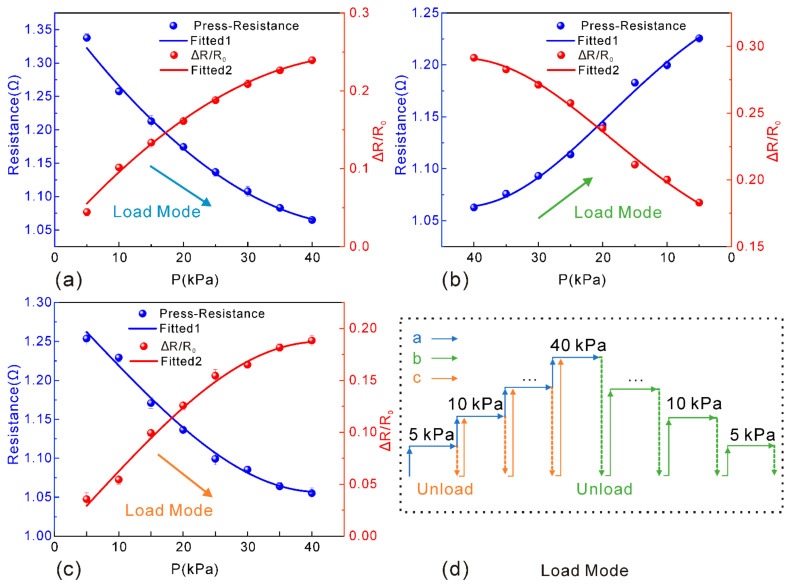
Change in resistance and sensitivity under different loading modes. (**a**) Resistance response performance with loading pressure from 0–40 kPa, maintaining loading after stabilization every 5 kPa without unloading each time; (**b**) Resistance response performance with loading pressure from 40 kPa down to 0, with data recorded until each stage of decrease of 5 kPa reaching stabilization, and with re-loading after each complete unloading; (**c**) Resistance response performance with loading pressure from 0 to 40 kPa at an interval of 5 kPa, and re-loading after each unload. (**d**) Schematic diagram illustration of different loading modes.

Figure 5 shows a diagram of the recorded data during three experiments with different loading approaches. As can be seen, the resistance change has a similar trend as the pressure loading for three loading approaches, and the error of resistance change for each loading approach will reach a stable value of 0.015 Ω. Additionally, the maximum sensitivity is 0.04 kPa^−1^, indicating that the sensitive layer of the RGO–AgNS–PDMS nanocomposite has rather good interfacial interactions with the substrate and the interdigital electrodes. Furthermore, the sensor reveals strong stability, and little fluctuation of resistance was observed when sudden loading was applied. By increasing the loading pressure, the resistance will be gradually decreased, because the conductive nanocomposite will be subjected to squeezing, thus leading to more internal conductive paths. By increasing the loading pressure to 40 kPa, the change in resistance tends to be saturated, indicating that an equilibrium of internal conductive paths can be established under compression. It is noteworthy that the sensitivity of the sensor is relatively small because of the low initial resistance. Moreover, resistance is negatively correlated with pressure, and the interval of relative change in resistance is also low. According to Equation (2), the sensitivity of the sensor is not very high, but it has good stability with high accuracy. Thus, the performance of the sensor can be applicable as a flexible wearable device.

### 3.6. Application

**Figure 6:**
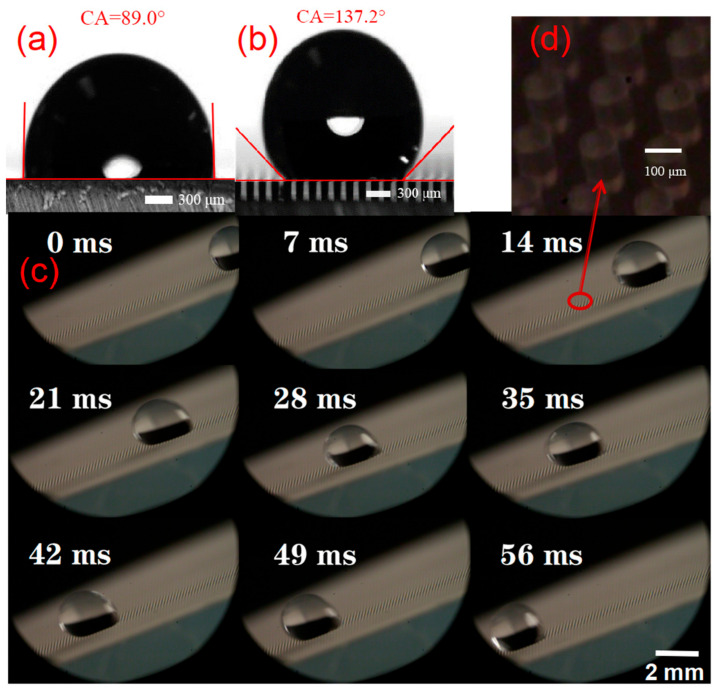
Characterization of hydrophobic wetting property of the microstructure surface of the sensor. (**a**) Contact angle measurement on the surface of ESSIL 296 without microstructure, scale bar = 300 μm; (**b**) Contact angle measurement on the surface of ESSIL 296 with micropillar array structure, scale bar = 300 μm; (**c**) Captured frames of a water droplet sliding on the tilting microstructure surface using a high-speed camera, scale bar = 2 mm; (**d**) Magnification of microstructure observation by the high-speed camera.

The influence of environmental factors should be avoided as much as possible in the application of sensors. The micropillar array structure of the prepared sensor surface not only has good sensing capability, but also endows its surface with hydrophobic performance. Figure 6 shows the contact angle measurement results with or without the micropillar array structure. From Figure 6a, it is clearly shown that ESSIL 296 has a certain hydrophobicity, but its hydrophobicity was not quite good, as water droplets with a contact angle of about 90° could still adhere to the surface without falling off. When a droplet of 10 μL was placed on the surface of the ESSIL 296 substrate with a micropillar array structure, the contact angle of water droplets could reach a contact angle of 137.2°. In essence, the micropillar array structure greatly enhanced the surface wetting property. Therefore, this microstructure presents wettability similar to the water striders, whose feet are covered with tiny hairs, enabling a super-hydrophobic wetting property [33]. When the surface is gradually tilted to about 45°, water droplets could roll off quickly. This process was successfully recorded with a high-speed microscopic camera. As shown in Figure 6c, the distance of a rapidly sliding water droplet on the surface of the micropillar array structure was 10 mm, and the duration was 62 ms, indicating that the surface of the sensor has good hydrophobic properties and can ensure good dustproof and self-cleaning effects in a long-run application.

**Figure 7:**
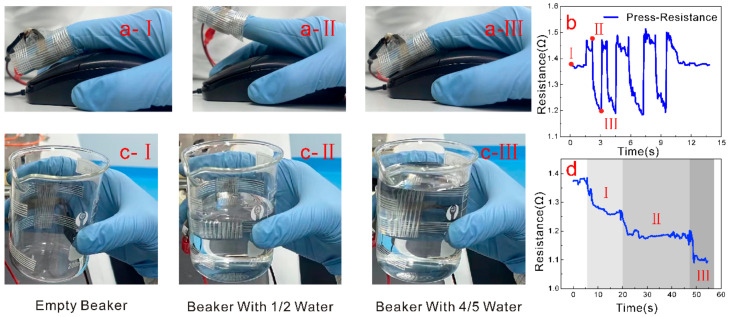
Demonstration and performance of the presented flexible pressure sensor mounted on a gloved finger. (**a**) Procedure of finger clicking the mouse, with I–III denoting the three stages of finger motions on the mouse; (**b**) Tested resistance of the sensor as the finger was clicking; b-I denoting the initial state of the finger, b-II denoting the finger lifting, and b-III denoting the finger clicking on the mouse, respectively; (**c**) Procedure of grasping a glass beaker, with I–III denoting varying amounts of water; (**d**) Result of resistance of the sensor indicating the stages of grasping the beaker with different amounts of water; d-I denoting holding of an empty beaker, d-II denoting holding of a beaker with 1/2 water in it, and d-III denoting holding of a beaker with 4/5 water in it.

The flexible piezo-resistive pressure sensor proposed is based on the polymeric nanocomposite of RGO, AgNS, and PDMS, thus endowing the sensor with good biocompatibility, flexibility, and tensile properties. Therefore, it can be worn as a kind of electronic skin on the irregular shape of joints of the human body or robots, to achieve the sensing of motion. Figure 7 demonstrates the performance of the sensor as mounted on a pointing finger of a glove to click the left key of the mouse or to hold on to a glass beaker. As shown in Figure 7a, the flexible pressure sensor was mounted to the finger by sticky tape, with the finger clicking the mouse naturally. During the repeated clicking process of the finger, the resistance of the sensor shows a change up and down, as is shown in Figure 7b. In addition, another demonstration was conducted for grasping the glass beaker using a hand to which the sensor was also applied (as shown in Figure 7c). As the quantity of water supplied to the glass beaker rose from zero to one-half to 4/5, the sensor resistance would gradually decrease with the increasing amount of water (Figure 7d). The above demonstration reveals the promising application potential of the presented flexible pressure sensors in wearable devices.

## 4. Conclusions

In summary, we present a novel type of flexible piezo-resistive pressure sensor with low resistance, stable sensing, and hydrophobic properties. The sensor was achieved by using a pressure-sensitive nanocomposite of RGO and AgNS in PDMS, thus leading to a uniform mixture and stable conductivity. The substrate of the sensor could be produced in batches by means of template imprinting and replication. Additionally, the flexible interdigital electrodes and sensitive functional layer can be prepared by blade-coating. As a result, the resistance of the pressure sensor was 1.6 Ω, the maximum sensitivity was 0.04 kPa^−1^, and the response performance of the sensor was stable even after 60 repeated experiments. The dynamic response time of the sensor was about 280 ms, and the recovery time was about 386 ms. Moreover, the micropillar array structure surface of the sensor has a good self-cleaning property. The prepared flexible pressure sensors are promising for potential applications in wearable electronics.

## Figures and Tables

**Table 1:** Comparison of different groups of composition for functional layer materials.

Number	Composition of Materials	Performance
1	RGO, PDMS	Good dispersion, poor conductivity
2	RGO, ESSIL 296	Poor dispersion, poor conductivity
3	AgNS, PDMS	Unstable and poor dispersion, good conductivity
4	AgNS, ESSIL 296	Poor dispersion, poor conductivity
5 *	RGO, AgNS, PDMS	Stable and good conductivity, good dispersion
6	RGO, AgNS, ESSIL 296	Poor dispersion, poor conductivity

* Selected composition for our study.

## Data Availability

Not applicable.

## Appendix A

In order to meet the needs of repeated production, the imprint molding method was used to fabricate the micropillar array structure and the interdigital electrode structure. ESSIL 296 was chosen as the template material because it has excellent toughness and resilience in harsh environments. The prepared template can be used to create the micropillar array structure and interdigital electrodes by repeatedly imprinting.

Briefly, the microstructure parameters of the micropillars was designed with a diameter of 100 μm and a height of 200 μm. The fabrication process is as follows. Figure A1a: a 3-inch silicon wafer was cleaned thoroughly, and photoresist AZ4620 was spin-coated on the wafer. Figure A1b: Prebaking of silicon wafer with photoresist on a 95 °C hot-plate for 10 min, prior to mask-aligned ultraviolet exposure of 365 nm wavelength for 33 s, post-baking for 5 min on a 100 °C hot plate, and developing by dipping into the sodium hydroxide (NaOH) aqueous solution with a mass fraction of 5‰. By this means, photoresist patterns can be obtained by rinsing in deionized water and blown dry with a nitrogen gun. Figure A1c: A round-hole array structure with a depth of 200 μm was etched on the developed silicon wafer by using an inductive coupling plasma (ICP)/reactive ion etching (RIE) process. Figure A1d: The excess photoresist on the silicon wafer was cleaned to obtain a round-hole array structure template on the silicon wafer. Figure A1e: The silicon wafer with etched structures was placed into an ICP180 etcher for hydrophobic process treatment by fluorocarbon polymer coating (C_4_F_8_). Figure A1f: Replicating with the etched silicon wafer as template by imprinting: ESSIL 296 precursor and curing agent with a ratio of 10:1 were mixed by stirring and degassed in a vacuum chamber, and then the prepared ESSIL 296 was supplied on the etched silicon wafer and placed again in a vacuum chamber for degassing, with a piece of glass covering and pressed, and placed in an 85 °C oven; Figure A1g: After curing for two hours, the sample of cured ESSIL 296 with micropillar array stuctures was peeled off the silicon template with microhole array structures. Figure A1h: The micropillar array structure of ESSIL 296 template was used to replicate the micro-hole array structure of ESSIL 296 and then cured in an 85 °C oven. Figure A1i: Hydrophobic treatment with C_4_F_8_, prior to peeling of ESSIL 296 microhole array template. The interdigital electrode mold can be fabricated using a similar procedure as mentioned above.
